# A unique ecological phenomenon quantified by hydroacoustics: the deep-sea eel spawning aggregation at Patience Seamount

**DOI:** 10.1098/rsos.260397

**Published:** 2026-10-28

**Authors:** Ben Scoulding, Sven Gastauer, Carlie Devine, Franziska Althaus, Alistair J. Hobday, Jessica Farley, Bruce Deagle, John Pogonoski, Rebecca Gorton, Ryan Downie, Alan Williams

**Affiliations:** ^1^ Environment, Commonwealth Scientific and Industrial Research Organization, Hobart, Tasmania, Australia; ^2^ NCMI, Commonwealth Scientific and Industrial Research Organization, Hobart, Tasmania, Australia; ^3^ Thünen Institute of Sea Fisheries, Bremerhaven, Germany

**Keywords:** optics, Australian marine parks, conservation, monitoring, orange roughy, *Hoplostethus atlanticus*, basketwork eel, *Diastobranchus capensis*

## Abstract

Patience Seamount, within Australia's Huon Marine Park, hosts persistent deep-sea fish aggregations dominated by basketwork eels (*Diastobranchus capensis*) and orange roughy (*Hoplostethus atlanticus*). Using integrated hydroacoustic surveys, deep towed camera imagery and physical sampling from 2023 to 2025, we quantified the spatial and temporal dynamics of these assemblages and produced species-specific biomass estimates. Acoustic backscatter attribution followed a two-phase approach: (i) camera-derived species distributions to inform spatial apportioning of backscatter and (ii) conducting biomass-constrained sensitivity analyses applying conservative bounds on orange roughy biomass (200–850 t) informed by historical assessments. We found a large, seasonally variable basketwork eel aggregation that increased markedly from March to May, when biomass reached 3547–3557 t (approx. 2.8 million individuals), likely corresponding to spawning. Distinct habitat preferences—with basketwork eels on deeper, flatter terrain and orange roughy on steep summit slopes—improved spatial separation of backscatter in this mixed-species environment. Without biomass constraints, orange roughy estimates became implausible relative to regional stock assessments, underscoring the need for species discrimination in deep-sea acoustic analyses. Patience Seamount's protection and recovery from historical fishing provide a rare opportunity for long-term study of deep-sea ecological processes. Our methods offer a scalable, non-invasive framework for monitoring deep-sea aggregations in marine protected areas.

## 1. Introduction

The deep sea, defined as depths below 200 m and encompassing over 95% of the ocean's volume, remains one of the least explored and most enigmatic environments. Characterized by low nutrient availability and limited primary production, it is recognized as an oligotrophic environment [1]. Nonetheless, it supports diverse and often highly specialized life forms, many of which exhibit broad dispersal ranges and elusive behaviours, making them inherently difficult to detect and study [2]. However, in certain regions and under specific conditions, large biological aggregations, typically associated with spawning [3–5] or feeding events [6,7], represent rare, ecologically significant phenomena. Seamounts, as prominent topographic features, are recognized as focal points for such aggregations, enhancing local productivity and biodiversity [8]. Historically, this richness has been exploited by fisheries targeting aggregations of species, such as orange roughy (*Hoplostethus atlanticus*), pelagic armourhead (*Pentaceros richardsoni*), alfonsino (*Beryx splendens*), roundnose grenadier (*Coryphaenoides rupestris*), cardinalfish (*Epigonus* spp.) and oreos (*Allocyttus niger* and *Pseudocyttus maculatus*), among others [9]. These practices have often resulted in severe population declines [9,10] and long-term ecological impacts on deep water coral reefs [11,12].

In response, marine protected areas (MPAs) have been declared in some regions to conserve deep-sea biodiversity [13,14]. Several of these deep-sea MPAs have demonstrated ecological and economic value by supporting species recovery and enhancing ecosystem resilience [15–18]. However, effective management requires an improved understanding of species distributions, ecological processes and habitat connectivity within areas where knowledge remains sparse [19]. Deep-sea aggregations may be in remote areas at extreme depths that are difficult to access owing to the substantial logistical and financial demands of deep-sea exploration. Consequently, many aggregations remain undocumented, and our understanding of their spatial and temporal dynamics is limited. Addressing these knowledge gaps is critical for the effective conservation and sustainable management of deep-sea biodiversity and biomass hotspots, such as seamounts, and for assessing ecosystem recovery following anthropogenic impacts.

Australia, with the world's third-largest Exclusive Economic Zone, has 62 Australian Marine Parks covering nearly 45% of its national waters (https://parksaustralia.gov.au/marine). Notably, 82% of this area lies below 1000 m, positioning Australia as a global leader in deep-sea conservation [20]. Established in 2007 as part of Australia's South-east Marine Park Network, the Huon Marine Park (figure 1) encompasses a series of seamounts off south-eastern Tasmania that were historically impacted by bottom trawling [10–12]. These seamounts support diverse deep-sea communities, including cold-water corals, sharks and fishes of high conservation value [12]. Among them, Patience Seamount (longitude 147.38° E, latitude –44.12° S, located 60 nautical miles southwest of Hobart, Tasmania, figure 1) is particularly notable as the site of the world's only known spawning aggregation of the deep-sea basketwork eel (*Diastobranchus capensis*).

**Figure 1.**
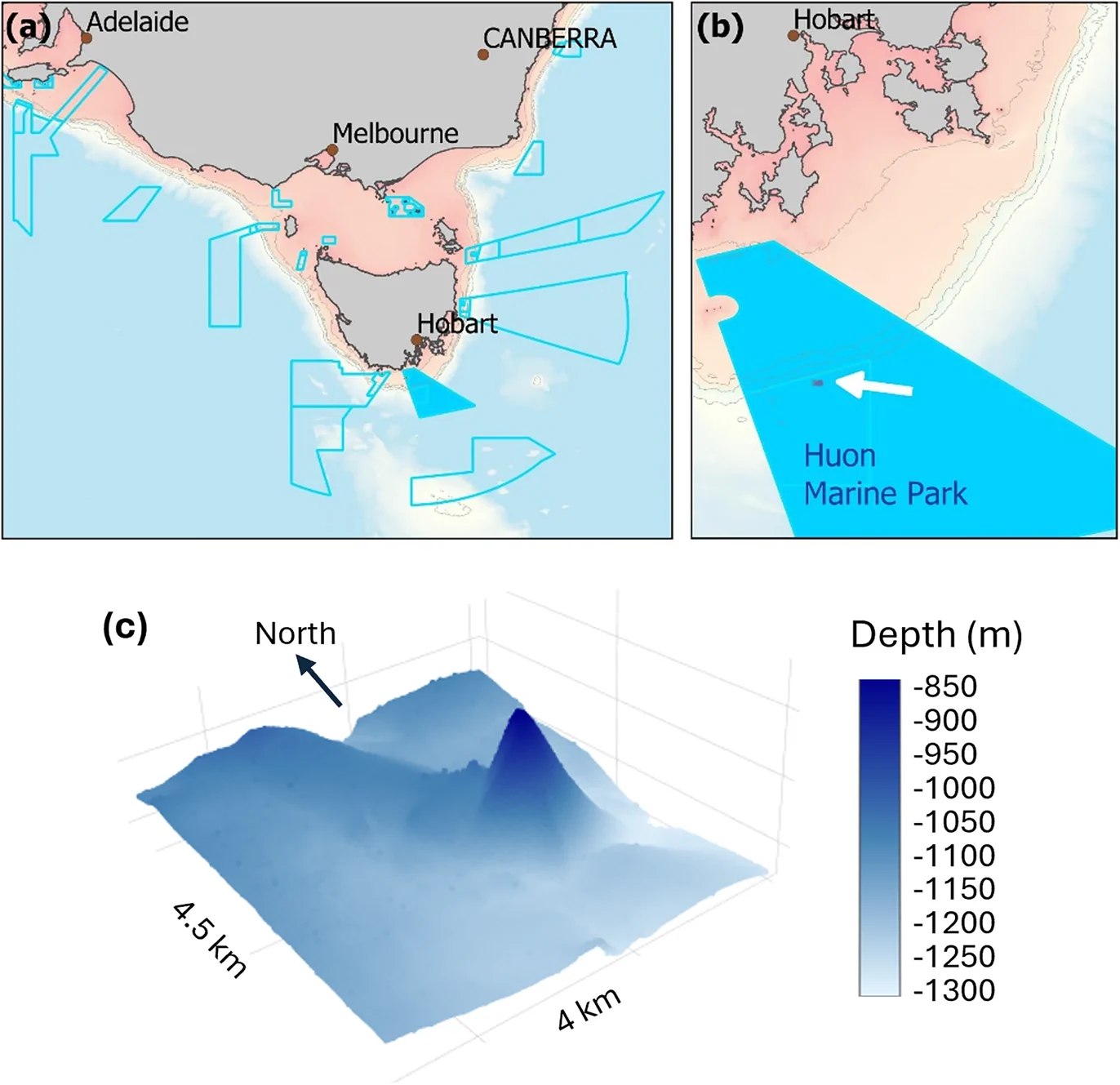
Location of Patience Seamount. (a) The South-east Marine Park Network, with individual parks outlined in light blue. (b) The Huon Marine Park, shown as a solid light blue polygon, corresponding to the highlighted area in (a). (c) Patience Seamount (longitude 147.38° E, latitude –44.12° S), indicated in (b) by a white arrow and small shaded area, is depicted here using a three-dimensional bathymetric map. Patience Seamount is a small, extinct volcanic feature (approx. 3 km² base area), rising to a peak depth of 857 m from a base of 1250 m.

True eels, comprising over 1000 species, including basketwork eels, are known to spawn in the ocean, yet the spawning ecology of most remains poorly understood [21,22]. Observations of anguillid eel aggregations are rare (exceptions include [23,24]) and absent in the deep sea, making the basketwork eel aggregation at Patience Seamount unique. This species is a large, ecologically important predator and scavenger, reaching lengths of up to 160 cm and occurring along the mid-continental slope (700–2000 m) across a broad distribution from the Southern Ocean to the Arctic, with the highest reported abundances around seamounts off Australia and New Zealand [25]. Patience Seamount is associated with a region of high acoustic backscatter, attributed primarily to basketwork eels [25]. However, key questions remain regarding the aggregation's biomass, its temporal persistence and whether it includes other species, namely orange roughy, and in what proportions.

In this study, we use a combination of hydroacoustic sensing, camera observations, and physical sampling to evaluate the composition, estimate the biomass and assess the seasonal persistence of the fish aggregation at Patience Seamount. Imagery is used to validate and partition acoustic data, and to explore environmental preferences, such as depth, slope and benthic habitat, of aggregated fishes. This study contributes to the global understanding of fish spawning aggregations as critical conservation features [5] and highlights the role of MPAs in supporting deep-sea species recovery [16]. Patience Seamount offers a rare opportunity to monitor conservation-led restoration in the deep ocean.

## 2. Methods

### 2.1. Survey programme

Three short biomass surveys were conducted aboard the 94 m research vessel (RV) *Investigator* in May 2023, March 2024 and April 2025 as it passed Patience Seamount on voyages to the Southern Ocean. Each survey lasted 10–12 hours and employed a star-pattern design [26], consisting of 12 to 20 evenly spaced, radially symmetrical 2 km long transects, starting at the peak of the seamount. Hydroacoustic data were collected using a calibrated Simrad EK80 echo-sounder [27] operated at 38 kHz (7° 3 dB beamwidth), mounted on a retractable keel 9.12 m below the surface. Narrowband pulses (power 1500 W and pulse duration 2.048 ms) were transmitted every 2 s, with data logged to 1500 m. Surveys were conducted at a vessel speed of around 10 knots.

Benthic imagery was collected using CSIRO's deep towed camera (DTC) system during two RV *Investigator* surveys, comprising one transect in March 2024 and two in April 2025. The DTC captured continuous high-definition video (60 frames per second) and stereo stills every 2 s. Lighting was provided by Quantum Strobes and DeepSea Power lights. Navigation was supported by ultra-short-baseline tracking and altimetry. The DTC was towed at approximately 1 knot, maintaining an altitude of 2–5 m above the seafloor along 2 km transects. A calibrated Simrad wideband transceiver (WBT) tube [27] with a vertically oriented 38 kHz transducer (18° 3 dB beamwidth) was mounted on the DTC to collect *in situ* target strength (*TS*, in dB re 1 m^2^) data for basketwork eels. *TS* is a measure of the acoustic energy backscattered by an individual and is needed to convert acoustic backscatter to abundance or biomass. The WBT Tube transmitted 0.256 ms narrowband pulses at 45 W every 0.2 s.

Six supplementary hydroacoustic surveys, designed to assess the seasonal persistence of the aggregation, were conducted aboard the 18 m training vessel (TV) *Navigator* in May, August and October 2023, and January, March and July 2024. A star-survey design with 8–16 transects (each 2 km in length) starting on Patience Seamount was followed. Data were collected using a Simrad WBT Mini echo-sounder connected to a dual-frequency 38/200 ‘Combi C transducer’ (13° longitudinal and 21° transverse 3 dB beamwidth), side-mounted 1.5 m below the surface. Only 38 kHz data were analysed, as the 200 kHz is useful only to a few hundred metres below the vessel owing to signal attenuation. Narrowband 38 kHz pulses (power 250 W and pulse duration 2.048 ms) were emitted every 2 s and recorded down to 1500 m depth, with the vessel moving at 5–6 knots. Although the WBT Mini can be calibrated and used to estimate biomass, limitations in data quality, signal-to-noise ratio and attenuation at low power, combined with its wide beamwidth, made this challenging at the depths being surveyed (>1000 m). As a result, this instrument was used qualitatively and was not used to estimate biomass.

Hook-and-line sampling was conducted during five of the six TV *Navigator* hydroacoustic surveys, supplemented by two additional fishing-focused surveys (May 2024 and April 2025), to validate species presence and to provide necessary bioinformatics. The sampling method for basketwork eels used a 10-hook squid-baited rig deployed to the seafloor during 20-minute sets. Moribund fish were euthanized with Aqui-S (150 mg l^–1^), stored on ice and later frozen at the CSIRO Hobart Marine Laboratories at –20°C, following approved animal ethics protocols.

### 2.2. Acoustic processing and echo-integration to estimate backscattering

Hydroacoustic data from RV *Investigator* and TV *Navigator* surveys were processed in Echoview version 14 [28]. Background [29] and impulse noise filters [30] were applied and data points outside the defined survey regions were excluded. Deadzone compensation followed Kloser [31], replacing near-bottom values with mean backscatter from 5 m above the seabed. Regions of high backscatter were inferred from 38 kHz echograms (figure 2a). Aggregations were identified based on depth, shape and texture.

**Figure 2.**
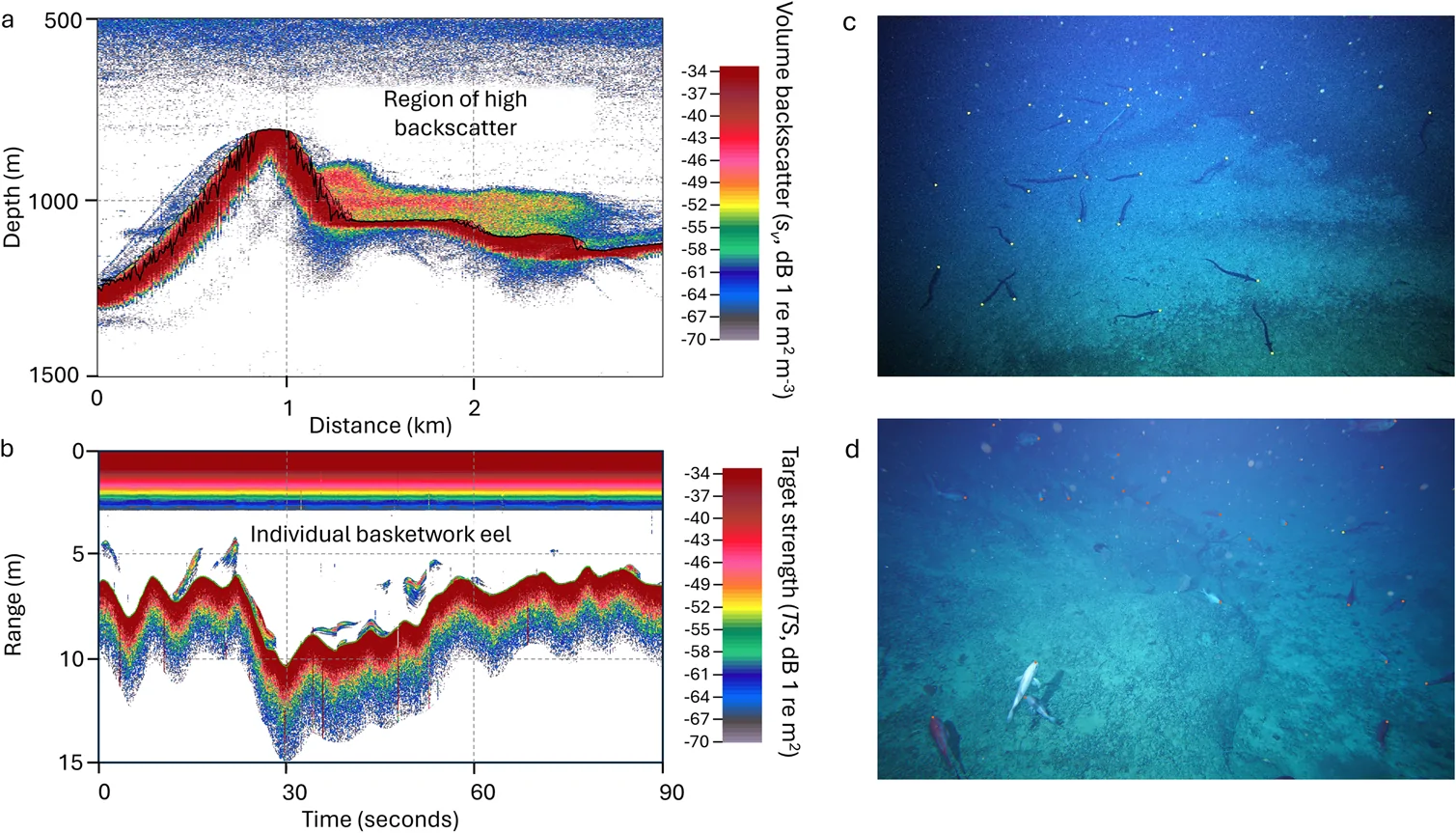
(a) Volume backscattering strength (*s_v_*, dB re 1 m^2^ m^–3^) echogram at 38 kHz collected in May 2023 on the RV Investigator showing a region of high backscatter near Patience Seamount, with an indication of the detected seafloor (black line) and (b) TS (dB re 1 m^2^) echogram at 38 kHz showing individual visually verified basketwork eels (*D. capensis*) recorded by an echo-sounder fitted to the DTC. Still images of (c) basketwork eels and (d) orange roughy (*H. atlanticus*) taken by the DTC during the RV Investigator survey in April 2025.

Volume backscattering coefficients (*s_v_*; dB re 1 m² m⁻³) from high backscatter regions were integrated (*s_A_*, m² nmi⁻²) from 10 m below the transducer to 2 m above the seabed. *s_A_* values were horizontally integrated over 100 m intervals. Biomass and uncertainty were estimated for the RV *Investigator* surveys using a stochastic partial differential equation (SPDE) framework and conditional Gaussian simulations in R using the gstlearn package [32], following methods described by Gastauer *et al.* [33]. The SPDE approach approximates continuous spatial random fields by projecting them onto a finite element mesh, enabling computationally efficient spatial interpolation and conditional simulations over large domains. The heavily zero-inflated acoustic data were transformed into a Gaussian distribution using an empirical anamorphosis model based on 800 Hermite polynomials. To account for the zero values, a truncation threshold was defined in the Gaussian space according to the proportion of zeros in the raw data. A Gibbs sampler (with 10 burn-in and 300 iteration steps) was then used to simulate the values below this threshold, conditional on the non-zero observations.

An omnidirectional variogram was computed for the truncated Gaussian variable and fitted with a model comprising a nugget effect and a Matérn covariance structure. Subsequently, 250 conditional Gaussian simulations were performed using the SPDE approach on a 25 m resolution grid covering the survey area. The mean of the simulations was back-transformed to the raw space using the anamorphosis model to estimate the area-backscattering coefficient (*s_A_*), and the coefficient of variation (CV, %) was computed from the simulations to assess spatial uncertainty.

The same approach was used to estimate *s_A_* distributions for the TV *Navigator* surveys.

### 2.3. Target strength estimates to convert acoustic backscatter to biomass

Hydroacoustic data from the DTC system were processed using Echoview version 14 [28], following Scoulding & Kloser [34]. Acoustic backscatter at 38 kHz (e.g. Figure 2b), still imagery (figure 2c and 2d) and video were synchronized using shared seafloor features to ensure spatial alignment. The seabed was detected using Echoview's ‘best bottom candidate’ algorithm, with manual corrections applied where needed. Impulse noise from the DTC altimeter was removed using a standard filter [30]. Single targets were detected with a –70 dB *TS* threshold and detections within 3 m of the transducer were excluded. Fish tracks, defined as sets of contiguous single-target detections, were manually classified as basketwork eels based on synchronized camera imagery (example shown in figure 2c).

To estimate the relationship between *TS* and length (*L* in cm, determined from stereo imagery, see below), a kernel-density-based curve fitting method was applied [35]. *TS* and length values were standardized, and 100 000 data points were sampled from a combined density function. A linear regression was fitted to the modelled data to estimate the slope and intercept of the *TS*–*L* relationship for basketwork eels. For orange roughy, the *TS*–*L* relationship was derived from an existing hybrid model, combining the slope from [36] and the intercept from [37].

### 2.4. Imagery processing and analyses

Imagery from the RV *Investigator* DTC tows in March 2024 (one) and April 2025 (two) were first processed using Marimba, a Python-based framework for managing marine imagery data [38]. The DTC data were used to: (i) count basketwork eels and orange roughy, (ii) obtain stereo length measurements of both species, (iii) proportion acoustic backscatter, and (iv) describe benthic habitats around Patience Seamount. In addition to data collected as part of this study, archived imagery from five DTC tows conducted on Patience Seamount during three earlier surveys in April 2007, April 2015 and December 2018 [25] was re-analysed to ensure consistency and enable comparison. Fish counts were conducted using imagery from the starboard-mounted still camera on the DTC, with frames analysed at 4-second intervals [25]. To avoid double counting, individual fish were tracked across consecutive frames. Measurements were made using EventMeasure (www.seagis.com.au), which enables three-dimensional length measurements from calibrated stereo image pairs. Only individuals within 10 m of the camera were measured. Measurements with a precision estimate, derived from the physical properties of the camera system and the three-dimensional intersection geometry that exceeded 5% of the measured length were excluded from analysis. To account for body curvature, each basketwork eel was measured using two or three straight-line segments from snout to tail. Each orange roughy was measured using a single straight line from snout to caudal peduncle.

For basketwork eels, a length–weight relationship (LWR) was modelled using the allometric growth equation:

$$\begin{array}{r} W=aL^{b}, \end{array}$$

where *W* is weight (g), *L* is length (mm), *a* is the scaling coefficient and *b* is the allometric exponent. Parameter estimates were derived from 27 specimens, seven collected during this study and 20 from Williams *et al.* [25]. For orange roughy, weight estimates were calculated using the published LWR from Lyle *et al.* [39]:

$$\begin{array}{r} W=2.07\times 10^{-2}\;L^{3.1238}. \end{array}$$

### 2.5. Proportioning acoustic backscatter to basketwork eels and orange roughy

We adopted a multi-phase approach to allocate integrated acoustic backscatter (*s_A_*) between basketwork eels and orange roughy. Firstly, species composition data were derived from video tows conducted during the March 2024 and April 2025 RV *Investigator* surveys. Species counts were aggregated across sequential 50 m segments along each tow. For each segment, the mean depth and geodesic distance from the peak of Patience Seamount were calculated, with distance measured from the shallowest point on the seamount to the end of each tow line.

Depth and distance data were binned into intervals (25 m depth bins from 850–1250 m; 100 m distance bins from 0–2000 m). Species proportions were calculated for each depth–distance bin and used to inform backscatter allocation. To assess spatial patterns, these proportions were integrated with gridded bathymetric data (approx. 15 m resolution) and aggregated into approximately 25 m spatial grid cells using rounded latitude and longitude coordinates. Each cell was assigned species proportions based on the nearest depth and distance bins. Depth- and distance-based proportions were then averaged to produce combined species distribution maps for basketwork eels and orange roughy. These maps were applied to allocate fractions of acoustic backscatter to each species. Species composition data from April 2025 were also applied retrospectively to the May 2023 survey for comparative analysis.

The relatively small *TS* of orange roughy (approx. 1/50 that of the basketwork eel) means there is a risk of overestimating orange roughy biomass when backscatter is integrated over a large area. To avoid overestimation, we imposed a conservative upper limit on orange roughy biomass informed by historical catch records. Patience Seamount was fished commercially for orange roughy between 1990 and 2003, yielding a total catch of 777 t across 570 trawl operations, with 96% of the catch taken during the first five years. Bycatch of basketwork eels was not reported. Based on current stock assessments for the eastern orange roughy stock [40,41], which includes Patience Seamount, we estimated pre-fishery biomass at Patience as approximately 842 t (777 t × 1.084 to account for depletion). Assuming recovery to 24.1% of virgin biomass [41], current biomass was estimated at approximately 203 t (approx. 114 000 individuals).

Based on historical catch records and stock assessments, we defined a plausible biomass range for orange roughy of 200 t (minimum) to 850 t (maximum). The upper bound of 850 t was based on the estimated pre-fishery biomass of orange roughy at Patience Seamount and therefore assumes that the current local biomass is unlikely to exceed unfished levels. To apply this constraint, we conducted an iterative sensitivity analysis that reapportioned acoustic backscatter between orange roughy and basketwork eels in 0.01% increments across the three surveys. For each increment, biomass was recalculated using species-specific *TS* and length–weight relationships. Reapportioning continued until orange roughy biomass estimates fell within the defined range. The remaining backscatter was attributed to basketwork eels and combined with eel-only estimates. This procedure ensured that biomass estimates were consistent with both historical exploitation patterns and contemporary stock recovery levels.

### 2.6. Classifying benthic habitat

Benthic habitats were continuously classified along each transect using a two-tiered system: (i) physical habitat, based on substrate type and bedform or relief, and (ii) biotic habitat, based on epibenthic fauna. Classification followed established protocols [11]. Substrate types were categorized using the CATAMI system [42], with refinements to distinguish coral rubble from other gravels. Bedform/relief classifications were paired with substrate types to describe seafloor structure. Biotic habitats were defined by aggregations of sessile fauna observed across multiple consecutive frames. Coral types were differentiated by morphology: stony corals formed matrix-like structures, while octocorals appeared as individual colonies. Habitat transitions were timestamped and georeferenced using 1-second navigation data.

### 2.7. Species preference across environmental gradients

Species preferences across slope, depth and habitat gradients were assessed using data from eight towed camera surveys (three current and five archival). Slope was calculated from interpolated depth profiles and geodesic distances between successive transect points, with the slope averaged over 50 m segments. Depth was derived from combined altimeter and depth sensor data.

To assess environmental preferences, species counts were binned by depth (25 m intervals), slope (5° intervals) and grouped by physical substrate group and biotic habitat type (according to the classification scheme described above). Preference scores were calculated for each category as the ratio of species observations to the total number of observations, with values >1 indicating conditions in which fish occurred more frequently than expected. *G*-tests of goodness-of-fit (R package DescTools [43]) were used to test for non-uniform species distributions. Where the *G*-test was significant, the significance of individual bins was then tested using approximated residuals and two-tailed *p-*values.

## 3. Results

### 3.1. Persistence and size of the high acoustic backscatter region (aggregation)

Hydroacoustic data were collected during nine surveys of Patience Seamount conducted between May 2023 and April 2025. These included three biomass surveys aboard the RV *Investigator* and six supplementary surveys aboard the TV *Navigator*.

Regions of high acoustic backscatter, indicative of fish aggregations, were detected in six of the seven surveyed months (figure 3). No such regions were observed in October 2023. High-backscatter regions were consistently located on the western flank of Patience Seamount, with no aggregations detected on the eastern side (figure 3).

**Figure 3.**
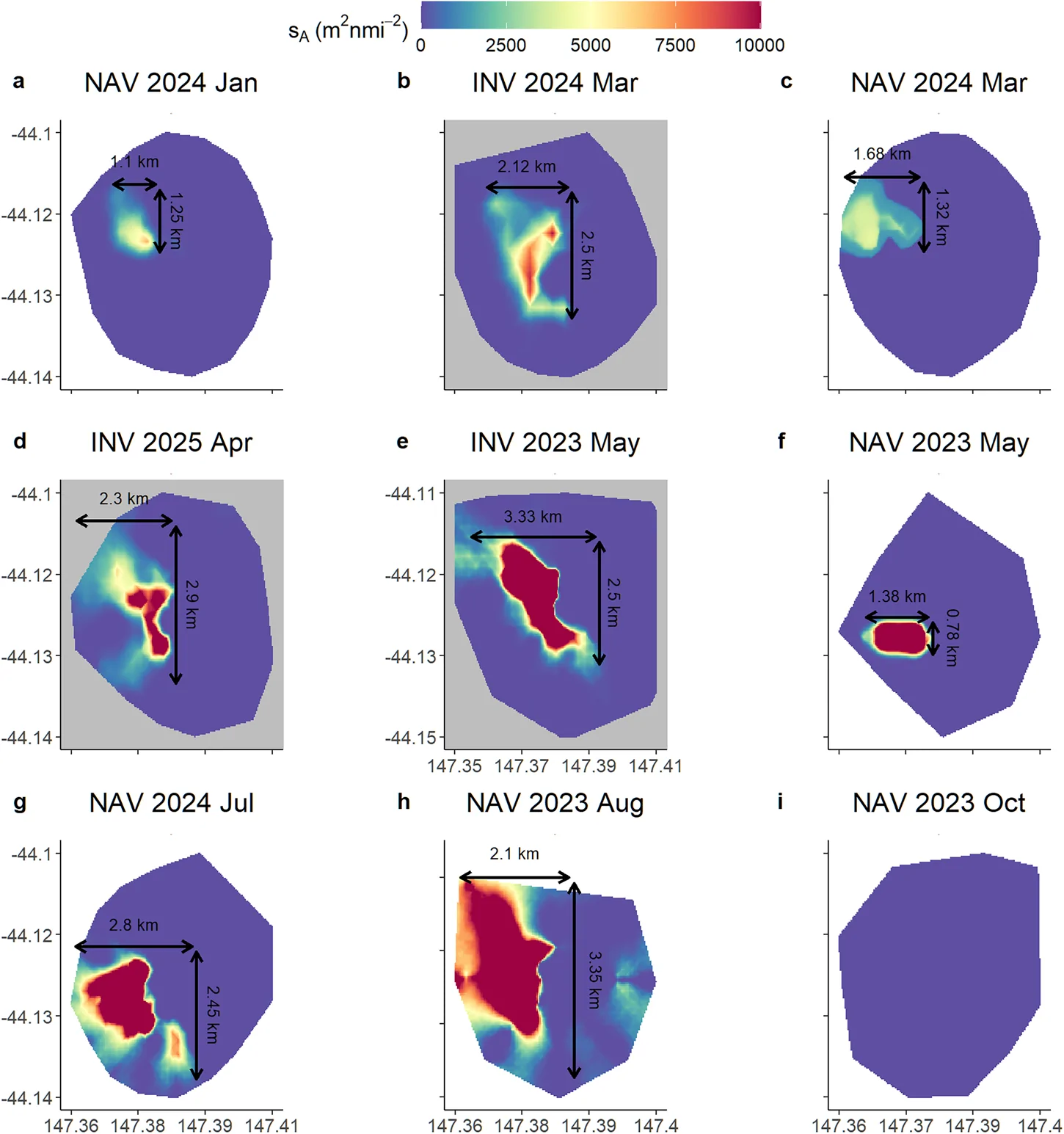
Mean distribution of the nautical area scattering coefficient (*s_A_*, m² nmi^–^²) from 250 conditional simulations for all hydroacoustic surveys conducted on the RV *Investigator* (INV) and TV *Navigator* (NAV). Arrows indicate the approximate maximum extension in easting and northing of the aggregation. Only RV *Investigator* data were calibrated. Individual maps (a–i) are sorted by month of the year.

The largest aggregation was recorded in May 2023 by the TV *Navigator*, spanning approximately 3.3 × 2.5 km horizontally and 150 m vertically (figure 3). The smallest aggregation was observed in January 2024, measuring 1.1 × 1.25 km horizontally. Overall, the size and acoustic intensity of the aggregation observed by the TV *Navigator* increased from January (equivalent area = 1.11 km^2^) to August (equivalent area = 4.70 km^2^) (table 1). Although the May aggregation was measured at only 0.89 km^2^ (table 1), we do not consider this its true size, based on the RV *Investigator* measuring the aggregation as 3.59 km^2^ in the same month and year. While the TV *Navigator* data are uncalibrated, limiting absolute comparisons, relative increases in backscatter suggest rising fish densities over this period (table 1).

**Table 1.** Average nautical area backscattering coefficient (*s_A_*, m^2^ nmi^–2^), size (*x* and *y* dimensions, km) and equivalent area (km^2^) of the acoustically detected aggregation at Patience Seamount. Surveys are ordered by month.

**vessel**	**month**	**year**	***s_A_* (m^2^ nmi^-2^)**	***x* dimension (km)**	***y* dimension (km)**	**equivalent area (km^2^)**
TV *Navigator*	January	2024	149	1.10	1.25	1.11
	March	2024	257	1.68	1.32	1.89
	May	2023	1070	1.38	0.78	0.89
	July	2024	2539	2.80	2.45	3.59
	August	2023	4682	2.10	3.35	4.70
	October	2023	-	-	-	-
RV *Investigator*	March	2024	538	2.12	2.50	3.39
	April	2025	817	2.30	2.90	2.66
	May	2023	2198	3.33	2.50	3.59

The size and acoustic intensity of the aggregation observed by the RV *Investigator* also increased by month between March and May (table 1). Notably, however, differences in aggregation size were observed between the RV *Investigator* and TV *Navigator* during near-concurrent surveys in March 2024 (figure 3b,c) and May 2023 (figure 3d,e). In both cases, the RV *Investigator* detected larger aggregations than the TV *Navigator*.

### 3.2. Bioinformatics

Across all TV *Navigator* surveys, 32 hook-and-line deployments yielded seven basketwork eel specimens. Although not detailed here, all captured basketwork eels were female, ranging in age from 5 to 22 years. The majority exhibited advanced vitellogenic oocytes, indicating maturity and readiness to spawn. Ovary organization was classed as synchronous, consistent with a determinate fecundity strategy. One individual caught in May 2024 showed post-spawning ovarian characteristics. All specimens had empty stomachs, supporting the conclusion that this was not a feeding aggregation.

A total of 121 basketwork eels were measured using stereo imagery from the DTC system collected during the RV *Investigator* survey in 2024. Owing to poor camera calibration in 2025, those data were excluded. Lengths ranged from 70.9 to 157.9 cm, with a mean of 101.4 cm. Based on the fitted LWR model (*a* = 0.00034, *b* = 3.29), the average 101 cm eel was estimated to weigh 1335 g. The model showed a strong fit with a coefficient of determination *R*² = 0.97.

For orange roughy, 100 individuals were measured in 2024. Lengths ranged from 29.7 to 53.3 cm, with a mean of 38.1 cm. Using the published LWR from Lyle *et al.* [39], the mean estimated weight for a 38 cm individual was 1782 g.

### 3.3. Target strength

Visual verification of acoustically detected basketwork eels using video and stereo stills from the 2024 DTC tow yielded a mean *in situ TS* of –33.14 dB re 1 m² at 38 kHz, based on 227 single-target detections from 17 individual eels and gave a *TS*–length relationship of:

$$\begin{array}{r} TS=136.09\;\log_{10}\left(L\right)-306.82. \end{array}$$

This equation estimates a *TS* of –34.6 dB re 1 m² for a 101 cm eel.

For orange roughy, the *TS*–length relationship was [36,37]:

$$\begin{array}{r} TS=16.37\;\log_{10}\left(L\right)-77.17. \end{array}$$

This gives a *TS* of –51.3 dB re 1 m² for a 38 cm orange roughy.

### 3.4. Distribution, abundance and species-specific allocation of acoustic backscatter based on video data

DTC video data collected during the RV *Investigator* surveys in March 2024 and April 2025 revealed distinct spatial distribution patterns for basketwork eels and orange roughy across Patience Seamount (figure 4). Depth- and distance-weighted proportions indicated that basketwork eels were dominant in deeper regions (>1050 m), with a broad distribution across the flanks and base of the seamount. By contrast, orange roughy were primarily concentrated near the peak and along steep slopes between 950 and 1100 m (figure 4). Heat maps of species proportions further showed that basketwork eels dominated areas located more than 1 km from the seamount peak, while orange roughy were largely confined to within 500 m of the peak.

**Figure 4.**
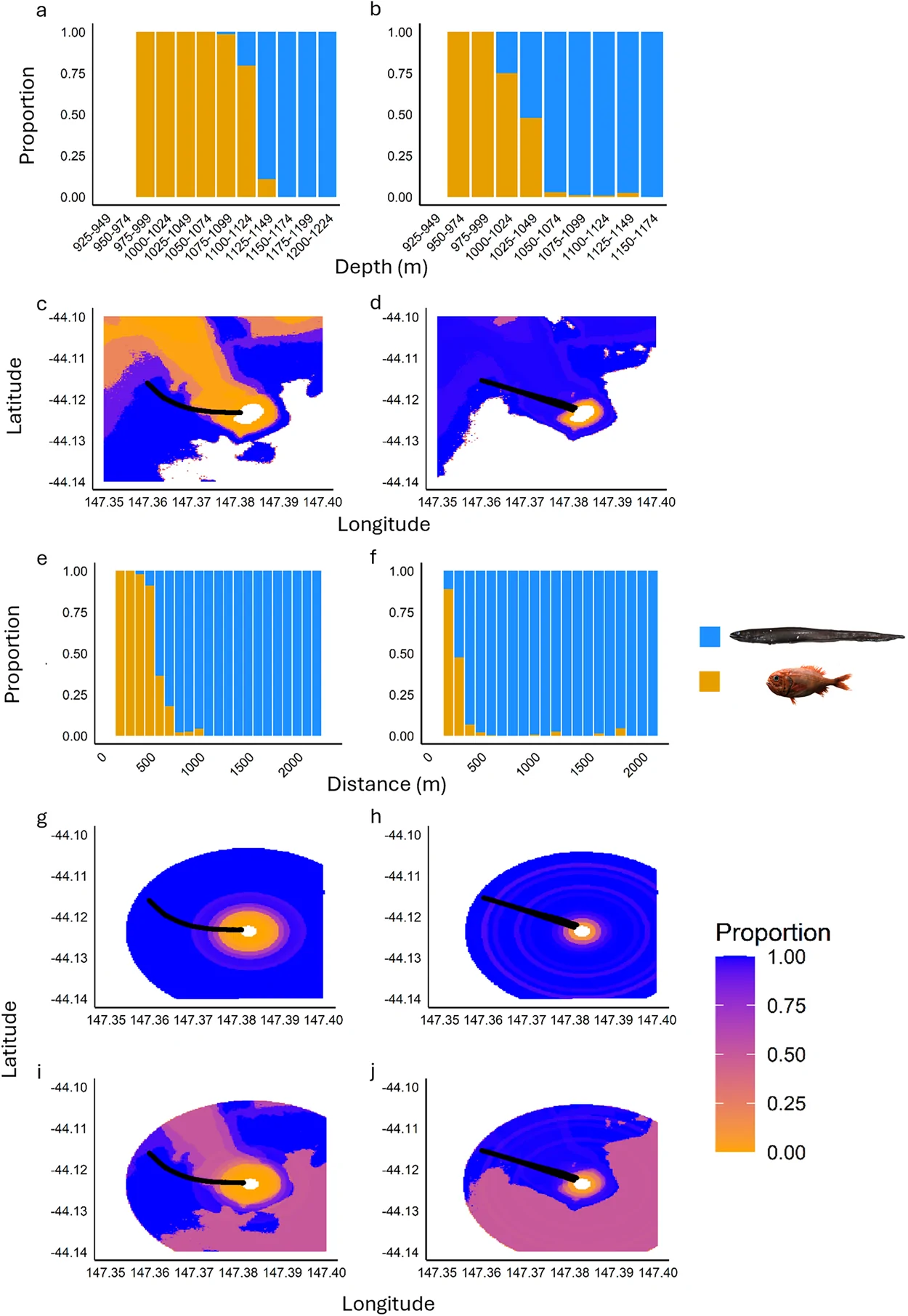
Proportions of basketwork eels (*D. capensis*, blue) and orange roughy (*H. atlanticus*, orange) observed at Patience Seamount based on imagery collected from one deep-towed camera transect in March 2024 (left column) and two transects in April 2025 (right column). (a,b) Species proportions by 25 m depth bins. (c,d) Heat maps of species proportions gridded on a 25-m-resolution bathymetric map. (e,f) Species proportions by 100 m distance bins from the peak of Patience Seamount. (g,h) Heat maps of species proportions by distance, overlaid on the 25 m bathymetric grid. (i,j) Combined heat maps showing averaged species proportions based on both depth and distance from the peak. The black lines in (c,d) and (g,j) show the position of the camera tows (the two April 2025 DTC tows overlap). Proportion values represent the relative biomass of basketwork eels to the combined biomass of basketwork eels and orange roughy. A proportion of 1 indicates 100% basketwork eels and 0% orange roughy, while a proportion of 0 indicates 0% basketwork eels and 100% orange roughy.

Temporal variation in species distribution and abundance was also evident between the two survey periods. In March 2024, orange roughy exhibited a broad spatial distribution, extending farther from the summit. By April 2025, their distribution had become more localized, and their abundance was markedly reduced (figure 4). By contrast, basketwork eels were more broadly distributed and occurred at higher densities in April 2025 compared with March 2024. During the March 2024 DTC tow, 1080 orange roughy were recorded—more than double the number of basketwork eels (457). However, across the two tows conducted in April 2025, a total of 3068 (96.3%) basketwork eels were observed, compared with only 117 (3.7%) orange roughy.

### 3.5. Biomass estimates of basketwork eels and orange roughy

Biomass estimates were derived from conditional simulations using the SPDE framework. A range of biomass estimates is presented for each survey based on the assumption that orange roughy biomass lies between 200 and 850 t. Biomass estimates consist of two components:

(i) eel-only biomass, calculated from backscatter attributed exclusively to basketwork eels (figure 5), and (ii) adjusted biomass, based on backscatter apportioned between orange roughy and basketwork eels according to the defined bounds for orange roughy biomass.

**Figure 5.**
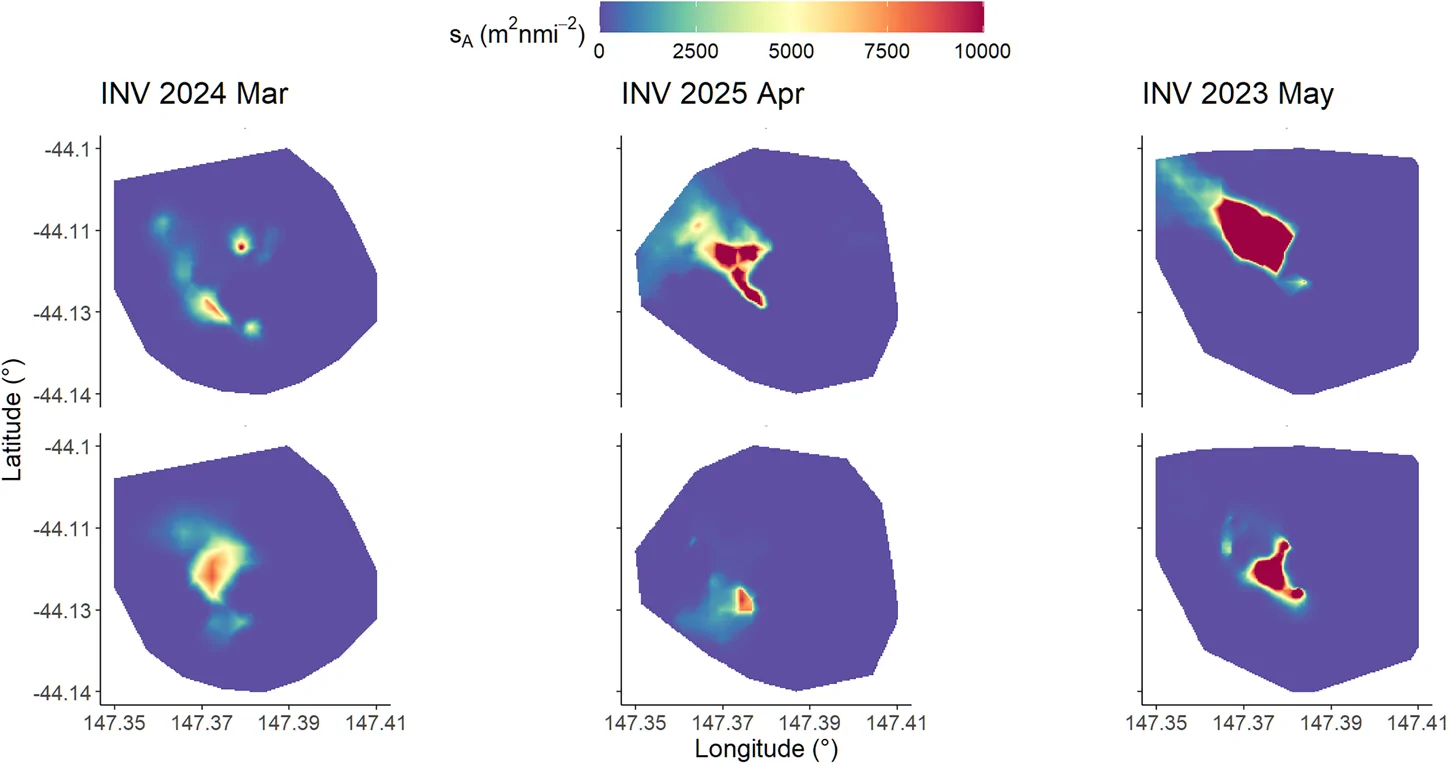
Mean distribution of the nautical area scattering coefficient (*s_A_*, m² nmi^–^²) from 250 conditional simulations for basketwork eel (*D. capensis*) (top) and orange roughy (*H. atlanticus*) (bottom) for the three RV *Investigator* biomass surveys in March 2024 (left), April 2025 (middle) and May 2023 (right).

For March 2024, the eel-only estimate was 265 t, corresponding to an abundance of approximately 0.21 million individuals (CV = 5.52%). When orange roughy backscatter was apportioned within the defined bounds, orange roughy accounted for 0.65–2.77% of total *s_A_*, while basketwork eels accounted for 97.23–99.35%, resulting in basketwork eel biomass ranging from 477 to 488 t (abundance approx. 0.36–0.37 million individuals, CV = 3.82%). Taken together, the total basketwork eel biomass for March 2024 was between 742 and 752 t.

For April 2025, eel-only biomass was 929 t (abundance approx. 0.74 million individuals, CV = 4.25%). Under constrained allocation, orange roughy accounted for 1.99–8.47% of backscatter, while basketwork eels accounted for 91.53–98.01%, giving a basketwork eel biomass between 147 and 158 t (abundance approx. 0.11–0.12 million individuals, CV = 6.51%). The total basketwork eel biomass for April 2025 was between 1076 and 1087 t.

For May 2023, eel-only biomass was 2716 t (abundance approx. 2.17 million individuals, CV = 5.97%). Under constrained allocation, orange roughy accounted for 0.38–1.61% of backscatter, while basketwork eels accounted for 98.39–99.62%, resulting in basketwork eel biomass ranging from 830 to 841 t (abundance approx. 0.62–0.63 million individuals, CV = 10.2%). Total basketwork eel biomass for May 2023 was estimated to be between 3547 and 3557 t.

These estimates show a pronounced increase in basketwork eel biomass from March to May, representing a more than 10-fold rise (table 2).

**Table 2.** Summary of basketwork eel (*D. capensis*) biomass and abundance estimates under two scenarios: (i) eel-only backscatter, and (ii) adjusted allocation where orange roughy (*H. atlanticus*) biomass was constrained between 200 and 850 t. For each survey, the table reports eel-only biomass and abundance, the range of adjusted eel biomass and abundance, total eel biomass (including eel-only component) and the percentage of acoustic backscatter (*s_A_*) attributed to basketwork eel.

**month**	**year**	**eel-only biomass (t)**	**eel only abundance (millions)**	**adjusted eel biomass range (t)**	**adjusted eel abundance range (millions)**	**total eel biomass range (t)**	**% *s_A_* attributed to eel**
March	2024	265	0.21	477–488	0.36–0.37	742–752	97.23–99.35
April	2025	929	0.74	147–158	0.11–0.12	1076–1087	91.53–98.01
May	2023	2716	2.17	830–841	0.62–0.63	3547–3557	98.39–99.62

### 3.6. Species habitat preference across environmental gradients

Basketwork eels and orange roughy were detected at depths ranging from 950 to 1250 m and 825 to 1250 m, respectively, with strong preferences within these ranges (figure 6). Basketwork eels showed significant preference for depths between 1050–1150 m, while orange roughy strongly preferred depths of 1000–1100 m. Both species were observed significantly less frequently outside these depth ranges.

**Figure 6.**
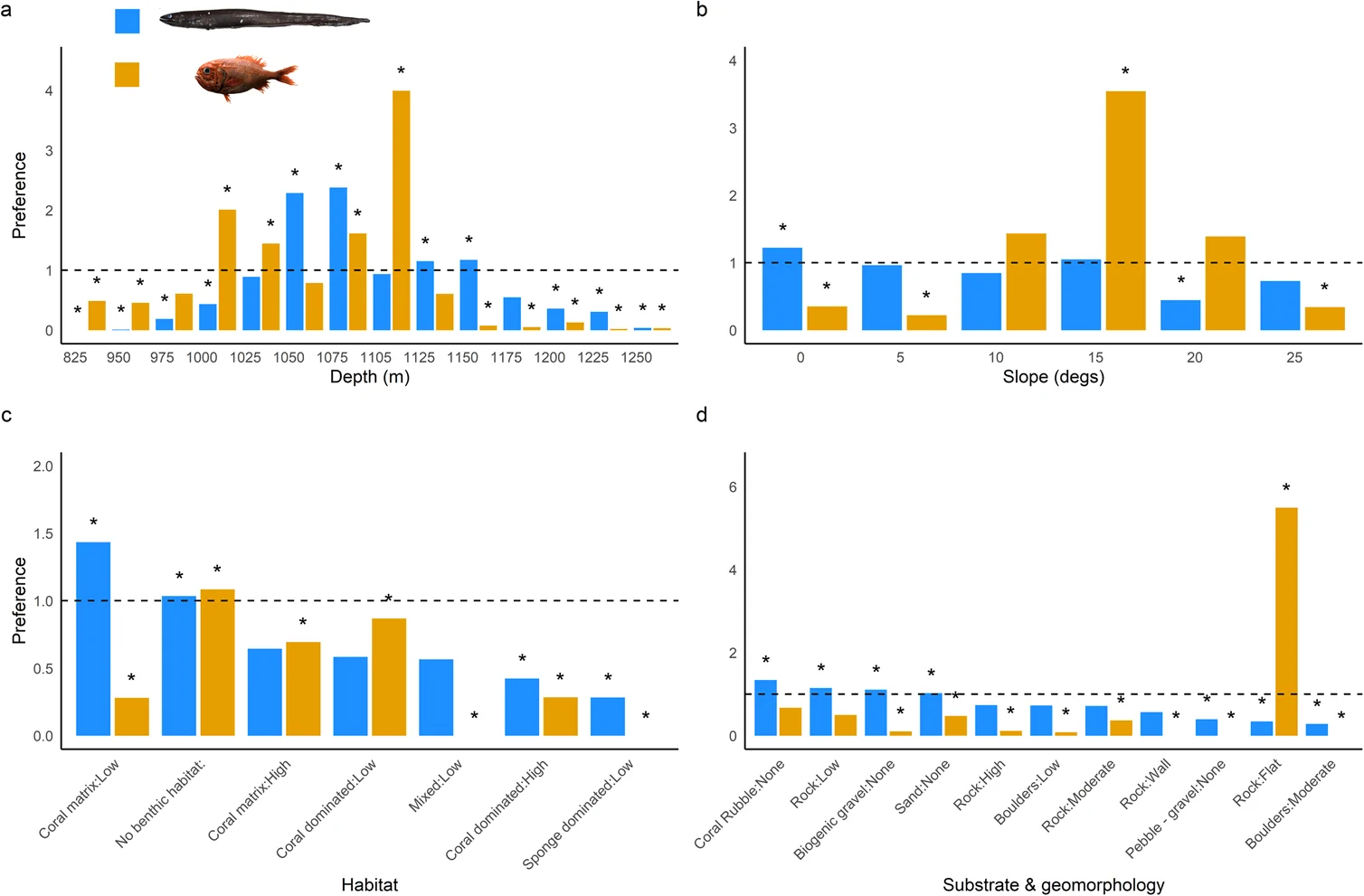
Environment preferences for basketwork eels (*D. capensis*) and orange roughy (*H. atlanticus*) at Patience Seamount, south-eastern Australia. Values greater than 1 (dotted line) indicate preference by a species, those less than 1 are less than expected values for the interval. (a) Depth preference in 25 m intervals. (b) Slope preference in 5° intervals. (c) Habitat preference by faunal group, ranked from highest to lowest for basketwork eels, (d) substrate and geomorphology preference by type, ranked from highest to lowest for basketwork eels. In each panel, significant (corrected *p*-values for the number of tests) preferences or avoidance are indicated by ‘*’, after a significant *G*-test result was returned for all eight distributions.

While depth preferences overlapped, basketwork eels exhibited a weaker but significant preference for flatter areas, whereas orange roughy strongly preferred slopes of approximately 15° and were under-represented in both flatter and steeper regions (figure 6b). Habitat preferences also differed by species. Basketwork eels preferred areas with low coral matrix and minimal benthic cover, avoiding regions with sponges and dense coral (figure 6c). Orange roughy similarly preferred areas with no benthic habitat and were less frequent than expected in all six other habitat types. Regarding substrate and bedform, basketwork eels showed a slight preference for four low-profile categories and were under-represented in the remaining seven (figure 6d). Orange roughy avoided a range of substrate types and showed a marked preference for flat rock, regardless of slope.

## 4. Discussion

Fish aggregations are commonly associated with seamounts worldwide, driven by interactions between physical structure, oceanography and ecological processes that enhance local productivity and biodiversity [8]. Patience Seamount, a compact 3 km² feature within the Huon Marine Park, hosts a persistent deep-sea fish aggregation [25]. Across nine surveys over 2 years, we integrated hydroacoustic data, camera imagery and physical sampling to assess its temporal persistence and species composition.

Hydroacoustic data indicated that the aggregation persisted from January to August, with no detectable signal in October. Spatial extent increased seasonally, peaking in August. Backscatter originated from both basketwork eels and orange roughy, with marked seasonal shifts in dominance. DTC imagery collected between March and May showed basketwork eels increasing steadily as orange roughy declined. Historical DTC data from April 2007 and 2015 reported high basketwork eel abundance and few orange roughy [25], consistent with April 2025 observations. Conversely, high backscatter recorded in December 2018 by Williams *et al.* [25] coincided with high numbers of orange roughy and fewer basketwork eels. Collectively, these data suggest that basketwork eels dominate from April to August, while orange roughy are more prevalent from December to March.

Although basketwork eels occur throughout the Huon Marine Park at low densities [25], Patience Seamount uniquely supports a dense seasonal aggregation. This is notable given comparable depth and oceanographic conditions on nearby seamounts. While habitat associations can influence deep-sea species distributions [44], they are often inconsistent [45], suggesting fine-scale ecological variability at Patience Seamount [46,47]. One possible driver is its proximity to the continental shelf, which may act as a physical boundary for bathyal migration or affect local circulation. As spawning in anguillid eels is influenced by currents and fronts [48], and seamounts play roles in eel reproductive ecology [22,49,50] unmeasured oceanographic features may contribute to aggregation formation.

Orange roughy aggregations occur across multiple Huon Marine Park seamounts, particularly during spawning (June–July) [25,51]. However, high orange roughy abundance outside this period indicates that Patience functions as a non-spawning aggregation site. Observed densities exceeded previous records [25], consistent with stock rebuilding [52] following historical overfishing [16,53]. Evidence of ecosystem recovery is further suggested by the presence of live *Aurgorgia tasmaniensis* on trawlable ground [54,55]. Patience thus represents an ecologically significant site for monitoring demersal fish dynamics and benthic recovery.

Imagery data collected during the RV *Investigator* surveys in March 2024 and April 2025 revealed clear spatial and temporal partitioning between basketwork eels and orange roughy across Patience Seamount. Basketwork eels were consistently dominant in deeper regions (>1050 m), broadly distributed across the flanks and base, and more prevalent at distances greater than 1 km from the peak. By contrast, orange roughy were concentrated near the peak and along steep slopes between 950 and 1100 m, with distributions largely confined to within 500 m of the summit. Temporal shifts in species dominance were also evident: orange roughy were more abundant and widely distributed in March 2024, while basketwork eel densities were markedly higher in April 2025, coinciding with a contraction in orange roughy distribution. These patterns align with species-specific preferences for depth and slope, with basketwork eels favouring deeper, flatter terrain and orange roughy preferring sloping areas near the summit. Neither species showed strong affinity for benthic habitat, instead selecting low-profile substrates with minimal biological cover.

These preferences, coupled with seasonal variation in abundance, provide a framework for interpreting hydroacoustic backscatter. The spatial separation and turnover between species suggest that physical variables, particularly depth and slope, offer a basis to objectively apportion backscatter, enabling confident species-specific biomass estimation. Biomass estimates were derived from backscatter data converted to proportion maps using depth–distance bins from one tow in March 2024 and two in April 2025. Orange roughy accounted for over half of the fish counts in March, consistent with their dominance near the summit, but this tow likely underestimated the basketwork eel's presence at depths between 1050–1150 m, missing areas later covered in April 2025. The April imagery showed that basketwork eels comprised 96% of observed individual fish, indicating a strong seasonal shift in dominance. This suggests that basketwork eels increasingly aggregate as they approach spawning, while orange roughy either disperse or are displaced. Owing to the absence of May 2023 video data, April 2025 proportions were used as a proxy.

Biomass estimates revealed a marked increase in basketwork eels from 742–752 t in March to 3547–3557 t in May. These estimates are based on a two-phase attribution of acoustic backscatter to basketwork eels. First, camera-derived species distributions were used to inform apportioning of integrated acoustic backscatter (*s_A_*) between basketwork eels and orange roughy along depth and geomorphic gradients on Patience Seamount, consistent with prior observations of a persistent, seasonally intensified basketwork eel aggregation at this site. Second, conservative bounds were applied to orange roughy biomass (200–850 t) informed by Eastern Zone stock assessments and recovery status, and *s_A_* was iteratively reapportioned at fine increments; this ensured local estimates remained plausible relative to regional dynamics. The upper bound of 850 t was based on the estimated pre-fishery biomass for Patience Seamount and therefore assumes that the present-day local population is unlikely to exceed unfished levels. Increasing the upper bound would result in additional acoustic backscatter being attributed to orange roughy and a corresponding reduction in basketwork eel biomass estimates. However, because the estimated biomass of basketwork eels substantially exceeded the plausible range for orange roughy across all surveys, the central conclusion that basketwork eels comprised the dominant component of the aggregation was robust to this assumption.

Without setting bounds on orange roughy, a naive attribution of all ‘orange-roughy’ backscatter to orange roughy yielded implausibly high biomass—29 565 (March), 12 460 (April) and 62 678 t (May). The May figure is especially problematic: it exceeds the regional spawning biomass for Tasmania's Eastern Zone reported in 2022 [41] and approaches approximately one-third of the estimated pre-fishery stock (approx. 200 000 t) [56], rendering such estimates unrealistic. This inflation is expected when basketwork eel signals are misclassified as orange roughy. Basketwork eels possess large and exceptionally elongate swim-bladders with a bulbous anterior section [25], yielding relatively high *TS* (*TS* ≈ –34.6 dB re 1 m²), whereas orange roughy lack a gas-filled swim-bladder and have tissue acoustic impedance close to seawater [57] (*TS* ≈ –51.3 dB re 1 m²). The approximatey 17 dB difference corresponds to an approximate 50× disparity in backscatter intensity: a 1 nmi² cell with *s_A_* = 100 m² × nmi⁻² attributed to orange roughy would imply >1 million individuals, whereas the same signal attributed to basketwork eels yields on the order of approximately 30 000 individuals. In mixed aggregations, failing to separate these signals inevitably leads to severe overestimation of orange roughy biomass.

The two-phase framework—camera informed spatial attribution followed by biomass-constrained sensitivity analysis—provides realistic bounds for eel biomass and mitigates orange roughy overestimation in mixed aggregations. This aligns local inference at Patience Seamount with regional stock dynamics and precautionary management, while highlighting the need to resolve species attribution where strong eel signals dominate. While our SPDE-based conditional simulations robustly capture the extreme spatial variability and zero-inflation inherent to the acoustic sampling of the aggregations, they do not propagate the underlying uncertainty of the *TS*–length and length–weight equations into the final biomass variance estimates. In this system, spatial sampling variance is highly pronounced and qualitatively expected to be the dominant source of uncertainty, heavily outweighing *TS* and LWR parameter variance. The use of an iterative sensitivity analysis to bound orange roughy biomass effectively restrains potential parameter-driven drift. Nonetheless, because these parameter uncertainties are omitted, our reported spatial variance estimates should be interpreted as conservative minimum bounds.

To improve accuracy, future surveys of this and any mixed-species spawning aggregations, should expand the spatial and temporal coverage of the DTC and incorporate multi-frequency [58–60] and broadband [61] acoustics. These technologies offer greater potential for species separation by exploiting anatomical differences [62] and could significantly enhance the reliability of biomass estimates in deep water environments. Furthermore, deep deployment platforms such as CSIRO's autonomous optical system [37] can overcome range limitations associated with higher-frequency echo-sounders (e.g. 120 and 200 kHz) but require commercial trawl gear, which is prohibited in most MPAs. Alternative approaches are therefore needed. With further refinement, such platforms, including autonomous technologies, may offer promising avenues for non-invasive deep water surveys. While deploying multi-frequency or broadband echo-sounders at depth can improve species discrimination, it also introduces calibration challenges, as transducers may shift performance under high pressure and low temperature [63]. In this study, the Simrad WBT Tube echo-sounder was calibrated at the surface, leaving uncertainty about its performance at depth. Future deployments should prioritize *in situ* calibration to ensure accuracy. Even with calibrated multi-frequency acoustics, camera imagery and physical sampling remain essential for species identification, size estimation and bioinformatics analysis [64].

The DTC system provided valuable data on horizontal distribution but offered limited insight into the vertical structure of the aggregation. High-backscatter regions extended up to 200 m above the seafloor in some months, prompting the use of a drop camera system in May and August 2023. These deployments confirmed basketwork eels occupying the water column up to 156 m above the seabed, with no orange roughy observed. The August deployment, coinciding with the largest backscatter region recorded (figure 5), indicated that high basketwork eel densities persist well beyond the assumed spawning peak in April–May. Although constrained by limited deployment opportunities, the drop camera proved effective. Future surveys should expand vertical sampling to improve biomass estimates and better understand aggregation structure [64–66].

Physical sampling remains essential for biometric data collection, yet deep-sea environments pose challenges. Hook-and-line fishing yielded only seven basketwork eels across 35 deployments. While the reproductive condition of these individuals was consistent with a spawning aggregation, the small sample size and the possibility of hook-and-line selectivity mean that further reproductive sampling across seasons, and including males, would strengthen this interpretation. Low catch rates may reflect reduced feeding activity during spawning (supported by empty stomachs), low fish densities or gear inefficiency. Mucus on lines suggested eels were taking bait but were not captured. To improve sampling success, especially in protected areas where trawling is prohibited, we recommend alternative methods, such as baited traps [67].

Despite these limitations, the study demonstrates that meaningful insights into deep-sea reproductive behaviour can still be achieved through non-invasive approaches. The methods outlined here provide a framework for investigating other fish and eel species suspected to form spawning aggregations, but which remain poorly understood [68]. Our findings show that acoustic and optical techniques are viable for monitoring deep-sea fish aggregations in MPAs. Patience Seamount emerges as a rare and ecologically significant site, offering a unique opportunity for long-term monitoring of basketwork eels, orange roughy and deep-sea habitats. This approach offers a scalable model for assessing other deep-sea aggregations with minimal environmental impact, underscoring the value of deep-sea MPAs and highlighting Patience Seamount as a natural laboratory for conservation-led restoration.

## Data Availability

Data and code are publicly accessible [69].
